# Sustainable Conversion of Coffee Ground Waste into Carbon Dots for Sensing Food Antioxidants

**DOI:** 10.3390/foods14223922

**Published:** 2025-11-17

**Authors:** Nan Jiang, Yuanjing Tao, Ruihong Wang, Xiaoran Zhao, Jingxuan Ren, Chenyang Jiang, Zihao Xu, Xuming Zhuang, Chao Shi

**Affiliations:** 1 School of Chemistry and Chemical Engineering, Yantai University, Yantai 264005, China; 2 Shandong Dyne Marine Biopharmaceutical Co., Ltd., Weihai 264300, China; 3 Shandong Dyne Financial Holding Children’s Pharmaceutical Co., Ltd., Weihai 264300, China; 4 Weihai Institute for Food and Drug Control, Weihai 264200, China

**Keywords:** low-cost, carbon dots, sensor array, total antioxidant capacity, coffee grounds

## Abstract

The total antioxidant capacity (TAC) of food products is a key parameter for assessing food quality and safety. In this work, iron-doped carbon dots (Fe-CDs) were successfully prepared using waste coffee grounds as a precursor with a satisfactory fluorescence quantum yield of 9.6%. The Fe-CDs exhibited exceptional peroxidase-like activity, which can oxidize colorless 3,3′,5,5′-tetramethylbenzidine (TMB) to form blue oxTMB. Concurrently, oxTMB induced an inner filter effect, quenching the fluorescence of Fe-CDs. After being added to antioxidants such as glutathione, ascorbic acid, and L-cysteine, the generated reactive oxygen species (ROS) are consumed, thereby preventing the oxidation of TMB. The color of the mixed solution changed from dark to light blue, accompanied by the fluorescence recovery of Fe-CDs. Nevertheless, these three antioxidants possessed remarkable differences in ROS elimination capability, which resulted in different signal responses in absorption and fluorescence, and were successfully used for constructing the colorimetric/fluorescent dual-channel sensor array. Furthermore, the sensor array signals were processed using principal component analysis to achieve simultaneous detection of glutathione, ascorbic acid, and L-cysteine, and were able to effectively discriminate between mixtures and individual antioxidants. The constructed sensor array was successfully applied for the TAC detection in various foods (including vegetables, fruit, and beverages) and for the precise differentiation of antioxidants in milk samples. Overall, the prepared sensor array exhibited outstanding potential in detecting food quality.

## 1. Introduction

Excessive levels of reactive oxygen species (ROS) in the body can accelerate aging and contribute to the development of various diseases [1,2,3]. Glutathione (GSH), cysteine (L-Cys), and ascorbic acid (AA), as common antioxidant molecules, play an irreplaceable role in maintaining cellular redox balance. Nevertheless, the limited antioxidants produced by the body are insufficient to exert a controlling effect on diseases, which have to draw support from the supplementation of exogenous antioxidants [4]. Thus, people began to consume large amounts of vegetables and fruits containing vitamin E and polyphenols in their daily diet to maintain redox balance within the body. However, excessive uptake of antioxidants may produce counterproductive effects and cause damage to human health [5]. Generally speaking, the antioxidant capacity of foods not only depends on the content of individual antioxidants but also requires a comprehensive assessment of the structural characteristics of various antioxidant components, as well as the possible interactions that may occur due to coexistence. Hence, it is of utmost importance to explore highly sensitive methods for detecting total antioxidant capacity (TAC) in order to evaluate the combined activity of all antioxidants in food.

To date, several methods have been used to detect TAC. For instance, chromatography enables sensitive and rapid quantification [6]. Electrochemical approaches facilitate portable detection [7]. However, the requirement for specialized personnel to operate the equipment limits its practical application. Spectrophotometry within spectroscopy encompasses ultraviolet-visible (UV-vis) and fluorescence (FL) spectroscopy. Due to its ease of operation and cost-effectiveness, it is widely favored by researchers and has seen extensive application in detection fields [8]. For example, Yang et al. developed a palladium/rhodium/iridium trimetallic octahedral nanoenzymatic colorimetric sensor for the determination of TAC in foodstuffs [9]. Liu’s team reported a nitrogen-doped carbon-supported Cu single-atom (Cu-SAC) nanozyme, which was used to study TAC in food and drugs by colorimetric analysis [10]. In addition, a study by He reported the successful application of MOF-derived Mn-doped NiO (Mn-NiO) oxidase mimics for the detection of TAC in tea samples [11]. However, most of the existing sensing systems rely on a single detection mode, which not only requires complex sample pre-treatment but also is prone to background interference, making them unsuitable for synchronous analysis of complex samples. The emergence of sensor arrays happens to provide brand new ideas and solutions to these challenges.

Inspired by the mammalian olfactory system, sensor array technology has emerged as an innovative solution. This strategy integrates multiple sensing units with cross-reactive responses and couples them with pattern recognition algorithms, enabling simultaneous identification and analysis of multiple target analytes [12,13]. However, conventional arrays usually rely on multiple sensing materials like different metal nanoparticles or fluorescent probes, which possess a complicated synthetic route and characterization method, and inter-element signal interferences may compromise reproducibility [14]. Consequently, developing high-performance sensor arrays based on a single multifunctional nanomaterial has become a critical challenge for balancing detection capability with system simplicity. Carbon dots (CDs), among various nanomaterials, offer a promising sensing element for building sensor arrays due to their unique optical properties, ease of functionalization, and other advantages [15].

Recently, biowaste-based CDs have attracted great attention in many fields due to their widespread sources, excellent optical properties, and enzyme-like activities [16,17]. Compared with the traditional organic matter as a precursor, using biomass waste to prepare CDs is a more environmentally friendly and economical way, which is in line with the concept of the circular economy. The CDs synthesized from corncobs via the hydrothermal method were reported to have a production cost of approximately $0.8 per gram—only one-quarter of the cost of using organic compounds as precursors [18,19]. Studies have been conducted to synthesize CDs from wastes such as banana peels [20], peach leaves [21], avocado skins [22], and other wastes. However, the carbon dots developed in these early works generally have low fluorescence quantum yields (QYs) (typically between 1.66% and 8.13%), which limits the sensitivity of their optical sensing applications to some extent. With the continuous growth of the coffee market, coffee has become one of people’s favorite beverages, and along with this comes over six million tons of waste coffee grounds. However, less than 10% of it is effectively reused, such as for the production of environmentally friendly materials and biofertilizers, etc. [23,24,25,26]. Delightfully, the high organic matter content of coffee grounds makes them a perfect source of carbon and nitrogen for CDs, and heteroatom doping can alter the internal electrical structure of CDs. Coffee grounds contain bioactive compounds like caffeine and chlorogenic acid, which can generate numerous functional groups on the surface of CDs and provide them with excellent water solubility [27]. Studies showed that the introduction of heteroatoms, especially metal ions, into CDs could regulate their physical and chemical properties by providing multiple electrons, unoccupied electron orbitals, and large atomic radii [28,29,30]. The physicochemical properties of CDs are usually improved by metal doping, such as enhanced stability, FL tunability, low toxicity, and especially increased peroxidase-like activity. This is because the doping of metal ions provides a new center of catalytic activity and raises the catalytic activity. For example, Zhou’s team used copper for doping to obtain CDs (Cu-CDs) that showed high peroxidase-like activity to detect thiols in serum [31]. Li’s team chose iridium as a dopant and prepared CDs with high peroxidase-like activity, which were successfully used to detect Hg^2+^ in real water samples [32].

**Scheme 1:**
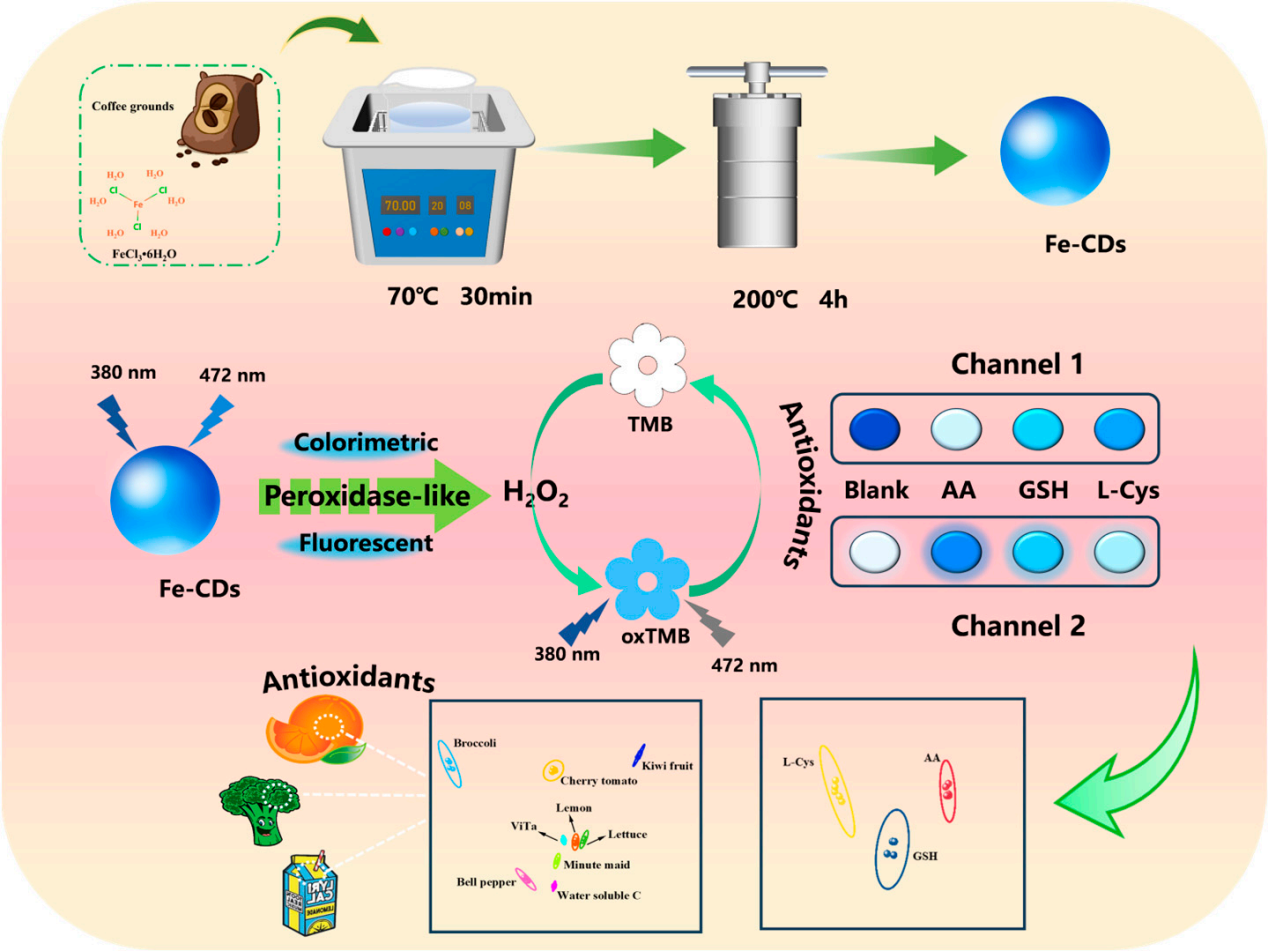
Synthesis process of Fe-CDs prepared by waste coffee grounds derivatisation, and schematic diagram of the construction of a dual-channel sensor array based on Fe-CDs for the detection of TAC in foods.

This study employed a hydrothermal method to successfully synthesize iron-doped carbon nanotubes (Fe-CDs) with peroxidase-like activity using waste coffee grounds as the precursor and ferric chloride as the dopant, exhibiting excellent fluorescence QYs. The detailed synthesis route and detection process are shown in Scheme 1. Fe-CDs catalyze hydrogen peroxide (H_2_O_2_) to oxidize colorless TMB into blue oxy-TMB, enabling visual detection. Detection revealed that H_2_O_2_ catalysis generated multiple ROS. Upon adding antioxidants (GSH, L-Cys, and AA), the blue Fe-CDs/H_2_O_2_/TMB mixture gradually faded, altering its UV-vis absorption spectra. Concurrently, the FL quenching of Fe-CDs caused by the internal filtering effect (IFE) induced by oxTMB is alleviated, allowing the Fe-CDs FL to recover. Leveraging the differences in the reducing capabilities of the three substances and their distinct response characteristics in absorption spectra and FL signals, a sensor array capable of detecting GSH, L-Cys, and AA was successfully constructed. This array can also precisely distinguish mixtures with varying proportions. In practical sample testing, the sensor array successfully identified and detected the three chemicals in fruits, vegetables, and commercial beverages, demonstrating significant application potential for enhancing food quality and safety.

## 2. Materials and Methods

### 2.1. Reagents and Instruments

Waste coffee grounds were collected from the brand of UNCLE BEAN. Iron (III) chloride hexahydrate was purchased from Sinopharm Chemical Reagent Co., Ltd. (Shanghai, China). GSH was obtained from Shanghai Macklin Biochemical Co., Ltd. (Shanghai, China). L-Cys and AA were purchased from Sigma-Aldrich (St. Louis, MO, USA). TMB and H_2_O_2_ (30%, *w*/*w*) were bought from Jilin China Science and Technology Co., Ltd. (Changchun, China). All other reagents are of analytical grade unless otherwise stated.

X-ray photoelectron spectroscopy (XPS) scanning curves were obtained on an ESCALAB 250Xi surface analysis platform (Thermo Electron, Waltham, MA, USA). The high-resolution transmission electron microscopy (HR-TEM) images were recorded on JEM-2020F (JEOL, Tokyo, Japan). The Fourier transform infrared (FTIR) spectrum was from 4000 to 400 cm^−1^ measured on an iCAN 9 spectrophotometer (Tianjin, China). The UV-vis spectra of Fe-CDs were measured using a TU-1901 UV-vis spectrophotometer (Puxi, Shanghai, China). The FL spectra of Fe-CDs were measured using an F-4700 FL spectrophotometer (JEOL, Tokyo, Japan). The ultrapure water used in the experiment is treated with ULUPURE (Chengdu, China). It should be noted that all experimental methods and characterization techniques in this study, although not mandatory to follow specific international standards (such as ASTM or EN), were designed and implemented in strict accordance with well-established and widely accepted practices in the field of nanomaterials and sensor analysis.

### 2.2. Preparation of Fe-CDs

The preparation of Fe-CDs was optimized with reference to the work of Zhu et al., specifically in the processing of precursors and the required mass [26]. A total of 2 g of waste coffee grounds was weighed and placed in an oven to dry and further milled using a mortar and pestle. The treated coffee grounds and 20.05 mg of Iron (III) chloride hexahydrate were mixed in 30 mL of ultrapure water and sonicated at 70 °C for 30 min. The mixture was transferred to a PTFE-lined autoclave and then reacted at 200 °C for 5 h. After cooling to room temperature, the resulting solution was centrifuged (8000 rpm, 10 min), and the supernatant was subsequently filtered through a 0.22 μm needle filter. The supernatant was subsequently filtered and dialyzed in a 1000 Da dialysis bag. Finally, the liquid was stored at 4 °C. For comparison with undoped carbon dots, pure CD (p-CDs) were synthesized following the same procedure as described above, except that Iron (III) chloride hexahydrate was not added.

### 2.3. Quantum Yield of Fe-CDs

Quinine sulfate exhibits a stable and well-defined fluorescence quantum yield in dilute acid solutions, rendering it widely employed as a standard reference for measuring fluorescence quantum yields. The procedure is as follows: five solutions with varying concentrations of both quinine sulfate and Fe-CDs were prepared, ensuring a gradient of UV-vis absorption peaks at the excitation wavelength of 350 nm. Under 350 nm excitation, the corresponding FL integral areas were recorded. A linear fit was then performed by plotting the integral areas against absorbance values, and the QY was ultimately calculated using Equation (1) [33].

(1) $$\Phi_{X}=\Phi_{ST}\left(\frac{k_{X}}{k_{ST}}\right)\left(\frac{\eta_{X}^{2}}{\eta_{ST}^{2}}\right)$$

where *Φ_X_* is the QY of quinine sulfate, *k_X_* and *k_ST_* are the slopes of the linearly fitted straight lines for Fe-CDs and quinine sulfate, respectively, and *η_X_* and *η_ST_* are the refractive indices of Fe-CDs (dissolved in distilled water with a refractive index of 1.33) and quinine sulfate (dissolved in a solvent of sulphuric acid with a refractive index of 1.33 at 0.1 M), respectively.

### 2.4. Peroxidase-like Activity and Kinetic Assay

The peroxidase-like activity of Fe-CDs was detected using TMB as a chromogenic substrate, which can be oxidized to blue oxTMB by ROS produced by Fe-CDs in H_2_O_2_ solution. Two sets of experiments were performed with different concentrations of TMB and H_2_O_2_:

(i) TMB (10 mM) + H_2_O_2_ (0.4, 0.6, 0.8, 1.0, 1.5, 2.0, 3.0 mM) + Fe-CDs;
(ii) H_2_O_2_ (50 mM) + TMB (0.4, 0.6, 0.8, 1.0, 1.5, 2.0, 3.0 mM) + Fe-CDs;

Different concentrations of TMB and H_2_O_2_ were mixed with diluted Fe-CDs, and HAc-NaAc buffer solution (pH 3.0) was added to bring the volume to a final of 3 mL with sufficient shaking. The sample was incubated at 50 °C for 10 min, and the UV-vis absorption peak at 652 nm was recorded immediately. The double reciprocal plot of the Michaelis–Menten equation, namely the Lineweaver–Burk plot, was utilized to determine two key kinetic parameters: the Michaelis constant (*Kₘ*) and maximum reaction velocity (*Vₘₐₓ*). Equations (2) and (3) correspond to the Michaelis–Menten equation and Lineweaver–Burk equation, respectively [34,35].

(2) $$V=\frac{V_{max}\times [S]}{K_{m}+[S]},$$

(3) $$\frac{1}{V}=\left(\frac{K_{m}}{V_{max}}\right)\times \left(\frac{1}{\left[S\right]}\right)+\left(\frac{1}{V_{max}}\right)$$

where *V* denotes the initial reaction rate and [*S*] denotes the substrate concentration.

### 2.5. Accurate Detection of GSH, L-Cys, AA

All data were collected under optimal conditions. The data of the constructed dual-channel sensor array were obtained from UV absorption spectra and FL emission spectra. The specific operation was as follows: 2.5 mL of HAc-NaAc buffer solution (pH 3.0), 50 μL H_2_O_2_ (5 mM), 50 μL TMB (1 mM), 50 μL of diluted Fe-CDs, and 50 μL of different concentrations of reduced small molecule solutions (GSH, L-Cys, AA) were added to the quartz cuvette. Afterwards, they were mixed thoroughly and incubated at 50 °C for 10 min, and the UV-vis absorbance at 652 nm was recorded. The FL signals were collected in the above operation. The FL spectral signals were recorded under excitation at 380 nm. The photomultiplier tube, slit, and scanning speed were 500 V, 10 nm, and 1200 nm/min, respectively. Five parallel experiments were performed for all the substances to be tested in the dual-channel sensor array, and the training “UV-vis/FL signal based on Fe-CDs × 3 reducing molecules × 5 concentrations × 5 replicates” was obtained. The data matrix was processed by Principal Component Analysis (PCA).

### 2.6. Detection in Real Samples

In order to evaluate the feasibility of the sensor array in practical applications, three types of food products were selected to simulate the complex mechanisms within foodstuffs, including fruits (lemon, kiwi, and sundried fruit), vegetables (lettuce, colored peppers, and broccoli), and beverages (Vitasoy, water-soluble C, and fruity oranges), were simulated and tested, and all of the above food products were purchased from local supermarket in Yantai, China. Fruits and vegetables were juiced, centrifuged at 8000 rpm for 10 min, filtered three times using a 0.22 μm filter membrane, and finally, the samples were stored at 4 °C away from light [36]. All beverages were filtered three times through a 0.22 μm filter membrane and stored under the same conditions as above. Thereafter, the actual samples were diluted to a 200-fold concentration, and 50 mL of the diluted actual samples was added for testing according to the assay procedure of 2.4.

## 3. Results

### 3.1. Characterization of Fe-CDs

**Figure 1:**
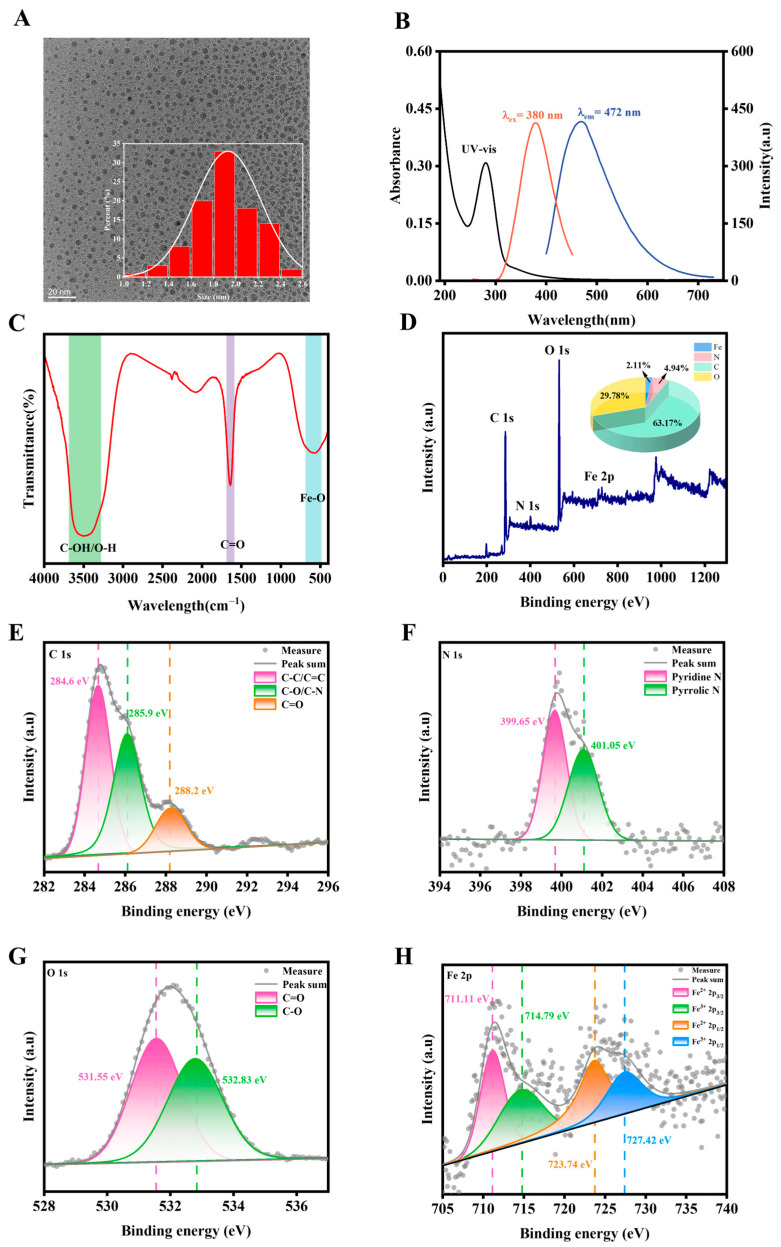
(**A**) The TEM image and the particle size distribution histogram (inset) of Fe-CDs. (**B**) The UV-Vis absorption spectrum (black line), excitation spectrum (red line), and emission spectrum (blue line) of Fe-CDs. (**C**) FTIR of Fe-CDs. (**D**) XPS full spectrum and elemental content (inset) of Fe-CDs. High-resolution XPS scanning spectra of (**E**) C 1s, (**F**) N 1s, (**G**) O 1s, and (**H**) Fe 2p of Fe-CDs.

HR-TEM was used to examine the morphology and size of Fe-CDs. According to Figure 1A, Fe-CDs showed a standard spherical shape and homogeneous dispersion in ultrapure water. The average particle size of the prepared Fe-CDs was 1.93 nm in the 1.0–2.8 nm particle size range. Furthermore, clear lattice fringes can be observed in the synthesized Fe-CDs from Figure S1, with a measured lattice spacing of 0.198 nm. The UV-visible spectra showed a distinct absorption peak near 280 nm for Fe-CDs (Figure 1B), which was due to the π→π* transition of the aromatic sp^2^ structural domain [33]. The strong fluorescent signal was captured at 472 nm under 380 nm excitation. The FL quantum yield of these Fe-CDs was calculated to be 9.6% using Equation (1) using quinine sulfate as a reference solution (Figure S2). As shown in Table S1, Fe-CDs possessed a considerable QY level compared to other carbon dots prepared by biowastes. To know the functional groups on the surface of Fe-CDs, FTIR spectra were measured in Figure 1C. Due to the decomposition or removal of weakly interacting functional groups during hydrothermal carbonisation (e.g., cellulose, chlorogenic acid, etc.), and the inherently weak signals from nitrogen-containing functional groups that are easily masked by strong background noise (such as C=O peaks), only a limited number of FTIR spectral peaks can be observed. The peaks at 3503 cm^−1^ were ascribed to the C-OH/O-H group. The peak at 1655 cm^−1^ is interpreted as a C=O stretching vibration. Moreover, the vibration at 587 cm^−1^ indicates the presence of Fe-O bonds within the Fe-CDs [34].

An XPS analysis was carried out to further understand the elemental composition and chemical state of Fe-CD. As shown in Figure 1D, the full spectra were mainly attributed to four typical peaks: C 1s (284.93 eV), N 1s (399.93 eV), O 1s (532.04 eV), and Fe 2p (711.33 eV). By calculating the full spectra peak area, the percentages of C, N, O, and Fe are 63.16%, 4.94%, 29.78%, and 2.11%, respectively. In Figure 1E, high-resolution XPS of the C 1s band was deconvoluted into four peaks at 284.6 eV, 285.9 eV, and 288.2 eV, which are assigned to C-C/C=C, C-O/C-N, and C=O bonds, respectively. As shown in Figure 1F, two peaks at 399.6 and 401.1 eV in the N1s spectra confirmed the presence of Pyridine N and Pyrrolic N atoms [35,37]. According to Figure 1G, it was possible to observe that the O 1s band of Fe-CDs could be deconvoluted into two peaks of C=O (531.55 eV) and C-O (532.83 eV), respectively. In addition, the Fe 2p spectra (Figure 1H) were fitted to four peaks at 711.1, 714.8, 723.7, and 727.4 eV, which were attributed to Fe^2+^ 2p_3/2_, Fe^3+^ 2p_3/2_, Fe^2+^ 2p_1/2_, and Fe^3+^ 2p_1/2_, respectively. These results indicated the successful doping of Fe and N, O elements in coffee grounds with the formation of functional groups.

### 3.2. Peroxidase-Mimicking Activity of Fe-CDs

**Figure 2:**
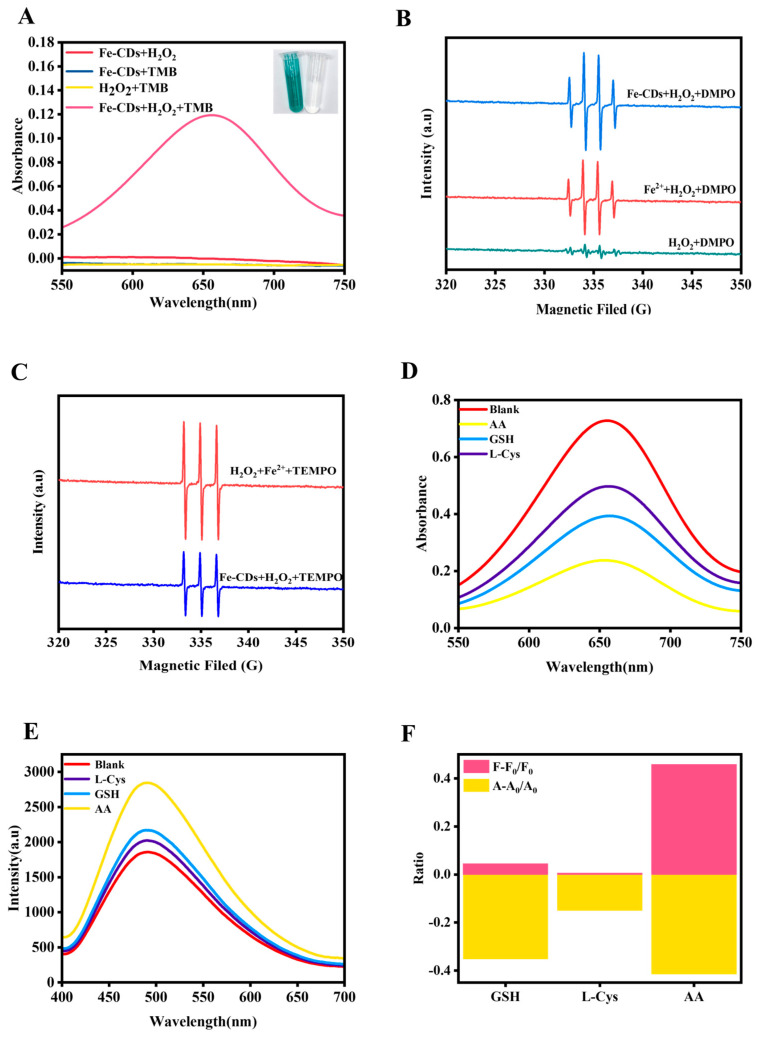
(**A**) UV-visible absorption spectra of TMB oxidized by Fe-CDs in H_2_O_2_ solution, along with the visual observation of colorless TMB (right inset) and blue oxTMB (left inset). EPR spectra of various Fe-CDs-based solutions after being incubated with (**B**) DMPO and (**C**) TEMPO. (**D**) UV-Vis absorption and (**E**) FL spectra of Fe-CDs after being incubated with various antioxidants. (**F**) Fingerprint pattern of GSH, L-Cys, and AA by colorimetric/fluorescent signals.

The peroxidase-like activity of Fe-CDs was evaluated by using TMB as a typical chromogenic substrate. In the presence of H_2_O_2_, TMB, and Fe-CDs, the mixed solutions were directly observed to change from colorless to blue oxTMB through naked-eye observation (inset of Figure 2A). When the mixed solution was analyzed by UV-vis spectroscopy, the results showed a characteristic absorption peak at approximately 652 nm [38,39]. In the absence of either H_2_O_2_ or Fe-CDs, no significant absorption peak was observed in the range of 550–750 nm (Figure 2A). Thus, Fe-CDs exhibited distinct peroxidase-like activity in catalyzing the oxidation of TMB. In contrast, the CDs mixed TMB solution remained colorless (Figure S3A), which indicated that the CDs exhibited negligible catalytic activity due to the lack of Fe-O moieties (Figure S3B). To further assess the catalytic performance of Fe-CDs, steady-state kinetic analysis was conducted under the optimized conditions (pH 3.0, 50 °C water bath) with a reaction time of 10 min (Figure S4). As shown in Figure S5A–D, it was calculated based on double inverse plotting that *K_m_* was 2.54 mM and *V_max_* was 12.75 × 10^−8^ M s^−1^ when TMB was used as the substrate, and *K_m_* was 1.27 mM and *V_max_* was 3.32 × 10^−8^ M s^−1^ when H_2_O_2_ was the substrate for the reaction. Compared with other transition metal-doped carbon dots, the Fe-CDs prepared in this work possessed excellent peroxidase activity, as shown in Table S2.

### 3.3. Mechanism of Catalysis

To clearly understand the catalytic process, the peroxidase-like activity catalytic mechanism of Fe-CDs was investigated. According to previous studies, the enzyme-mimicking catalytic mechanism primarily involved the formation of ROS [23,40]. Consequently, electron paramagnetic resonance (EPR) spectroscopy was employed to analyze the types of ROS generation during the catalytic process. 5,5-Dimethyl-1-pyrroline N-oxide (DMPO) can serve as a spin-trapping agent for capturing both superoxide anion radicals (O_2_^•−^) and hydroxyl radicals (•OH), while 2,2,6,6-tetramethylpiperidinyl-1-oxide (TEMPO) is capable of capturing singlet oxygen (^1^O_2_). As shown in Figure 2B, the group of Fe-CDs and Fe^2+^ in H_2_O_2_ solution displayed remarkable •OH signal while the control group showed an insignificant signal, this is because the control group lacks Fe-CDs (which provide Fe-O active sites to activate H_2_O_2_ decomposition) and H_2_O_2_ (as oxidant) simultaneously, failing to generate •OH and thus no distinct EPR signal of •OH-DMPO adduct. Analogously, as illustrated in Figure 2C, the EPR signal of Fe-CDs in H_2_O_2_ solution after being incubated with TEMPO showed a significant ^1^O_2_ signal, because TEMPO specifically traps ^1^O_2_ to form a characteristic adduct, which exhibits a distinct 1:1:1 three-line EPR signal under test conditions, directly confirming Fe-CDs catalyze H_2_O_2_ to generate ^1^O_2_. Therefore, the ROS generated by Fe-CDs-catalyzed H_2_O_2_ decomposition were identified as •OH and ^1^O_2_, providing conclusive evidence that Fe-CDs possessed exceptional peroxidase-like activity.

### 3.4. Detection of Antioxidants Based on the Dual Channel Sensor Array

Following the successful production of Fe-CDs with peroxidase-like activity, their enzymatic activity was utilized to build a dual-channel sensor array employing colorimetric/FL. The detection ability of the sensor array was examined by three common antioxidant substances in the human body, including GSH, L-Cys, and AA. As the natural enemy of ROS, these three antioxidants were able to deplete the •OH and ^1^O_2_ produced by Fe-CDs catalyzing H_2_O_2_, which ultimately affected the oxidation degree of TMB. As a consequence, the oxidation level of TMB shows remarkable differences due to the different reductive capabilities of the three antioxidants, and then shows the difference in solution color. To verify the aforementioned conjecture, the ultraviolet-visible spectra tastings were conducted after being individually added three antioxidants of the same concentration. As illustrated in Figure 2D, the maximum absorption peaks were mainly observed at 625 nm. The addition of the three antioxidants significantly reduced the peak intensities compared to the blank group, and there was no overlap in the intensity of the absorption peaks with each other. The reacted solutions were also subjected to FL detection, and it was found that the FL intensity of Fe-CDs decreased to different degrees (Figure 2E). The reason for the decrease in FL intensity could be attributed to the fact that the absorption spectra of the blue oxTMB were located at 370 nm, which has a lot of overlap with the excitation spectra of Fe-CDs (λ_ex_ = 380 nm), which triggered an internal filtering effect (Figure S6). Meanwhile, the p-CDs were also subjected to the above experiment, and it was found that there was no change in the FL of p-CDs before and after the reaction. Similarly, the FTIR test showed that the surface groups of p-CDs had no change before and after the reaction (Figure S7). Similarly, the FTIR test showed that the surface groups of p-CDs had no change before and after the reaction (Figure S8). Therefore, p-CDs were excluded as sensing elements for the detection of antioxidants. In summary, Fe-CDs possessed the recognition capability for three common antioxidant molecules, which confirmed the feasibility of the constructed colorimetric/fluorescent sensor array. As shown in Figure 2F, the fingerprints generated for the three antioxidants also confirmed the feasibility of the sensor array.

**Figure 3:**
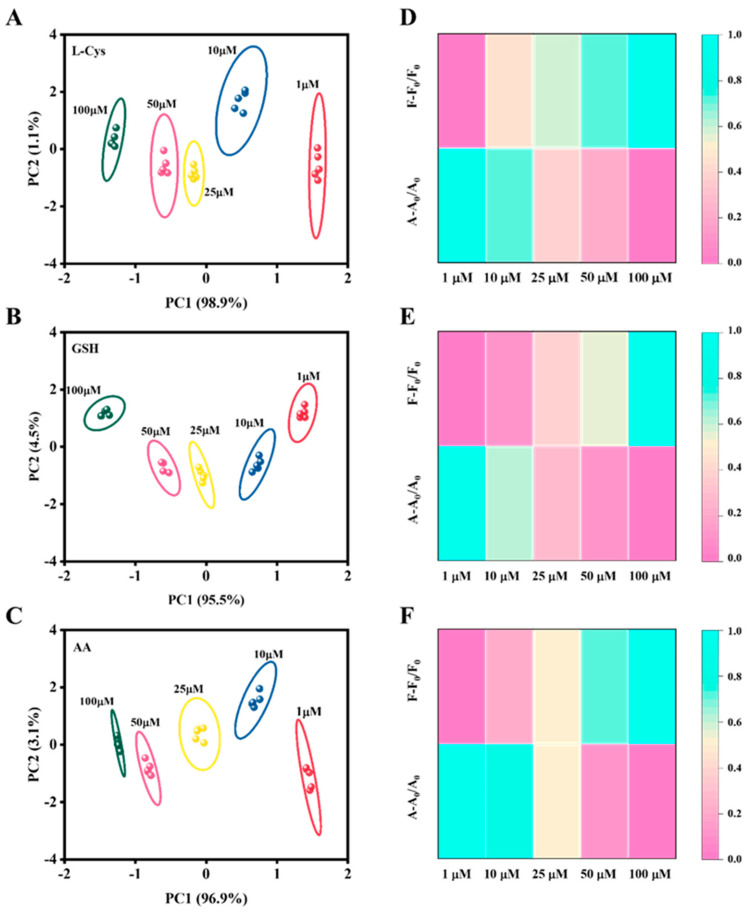
PCA plots of the sensor array corresponding to different concentrations of antioxidants. PCA plots for varying concentrations of (**A**) L-Cys, (**B**) GSH, and (**C**) AA. Heatmaps corresponding to different concentrations of (**D**) L-Cys, (**E**) GSH, and (**F**) AA.

Then, the collected signals were subjected to PCA. As shown in Figure 3A–C, the sensor array was able to form a separate confidence ellipse and accurately identify different concentrations of a single antioxidant; the same results were obtained by heat maps (Figure 3D–F). Crucially, the sensor array’s capability for qualitative identification was rigorously tested by analyzing the three antioxidants at identical concentrations. The PCA score plots in Figure S9A–E present a clear spatial separation of the clusters corresponding to GSH, L-Cys, and AA across a wide concentration range (1–100 µM). The confidence ellipses for each antioxidant are well-isolated with no significant overlap, indicating that the unique “fingerprint” response generated by each analyte is highly reproducible and distinct. This high-fidelity discrimination stems from their differing reducing capacities and kinetic interactions with the Fe-CDs/H_2_O_2_/TMB system, which in turn produces characteristic signal patterns in both the colorimetric and fluorescent channels. The progressive shifts in the PCA clusters with increasing concentration further underscore the dynamic and concentration-dependent nature of the sensor array’s response. Although the absorbance of oxidized TMB and the fluorescence of Fe-CQD are highly correlated overall (primarily driven by the internal filtering effect), the two-dimensional PCA plot still clearly distinguishes them when analyzing antioxidant mixtures. The fundamental reason lies in the fact that within the mixture system, these two signals undergo decoupling through multiple mechanisms. This generates new variance independent of the principal effect (PC1), captured by the second principal component (PC2), ultimately manifesting as spatially separated clusters in the plot. These results collectively confirm that the developed dual-channel sensor array possesses robust capabilities for the simultaneous identification and differentiation of these three structurally similar antioxidants.

**Figure 4:**
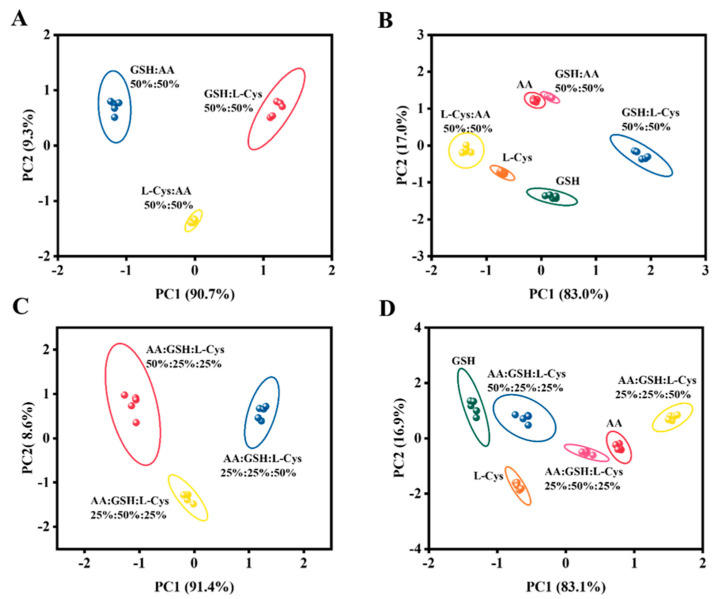
PCA plots for different scales and levels of mixed antioxidants. (**A**,**B**) Binary mixtures of different types of antioxidants are analyzed and distinguished from individual antioxidants. (**C**,**D**) Analytical discrimination between ternary mixtures of various antioxidants and their components.

To further investigate the analytical and recognition abilities of the fabricated sensor array for multiple mixtures, the PCA detection of mixing GSH, L-Cys, and AA was conducted. Firstly, the binary mixtures of GSH/AA = 1:1, GSH/L-Cys = 1:1, and L-Cys/AA = 1:1 (all at concentrations of 50 μM) were tested. As illustrated in Figure 4A, the sensor array remained capable of accurate identification even in binary mixtures. Simultaneously, the signals of 50 μM GSH, L-Cys, and AA were processed together (Figure 4B), yielding well-separated and non-overlapping results. Although the AA response was close to the mixture clusters, it remained distinguishable. Meanwhile, as shown in Figure S10, the heatmap of the binary mixture also reveals the differences between them. Ulteriorly, the PCA plots still demonstrated excellent discriminative ability when testing the ternary mixtures at (AA/GSH/L-Cys = 1:1:2, 1:2:1, and 2:1:1, all at 50 μM) (Figure 4C,D). Additionally, the color variations in the heatmap further highlighted the outstanding differentiation capability of the sensor array (Figure S11). Thus, it was demonstrated that the developed sensor array could effectively differentiate between three common antioxidants in food products, thus providing a new approach to the detection of TAC in food products.

### 3.5. Identification of Actual Samples

**Figure 5:**
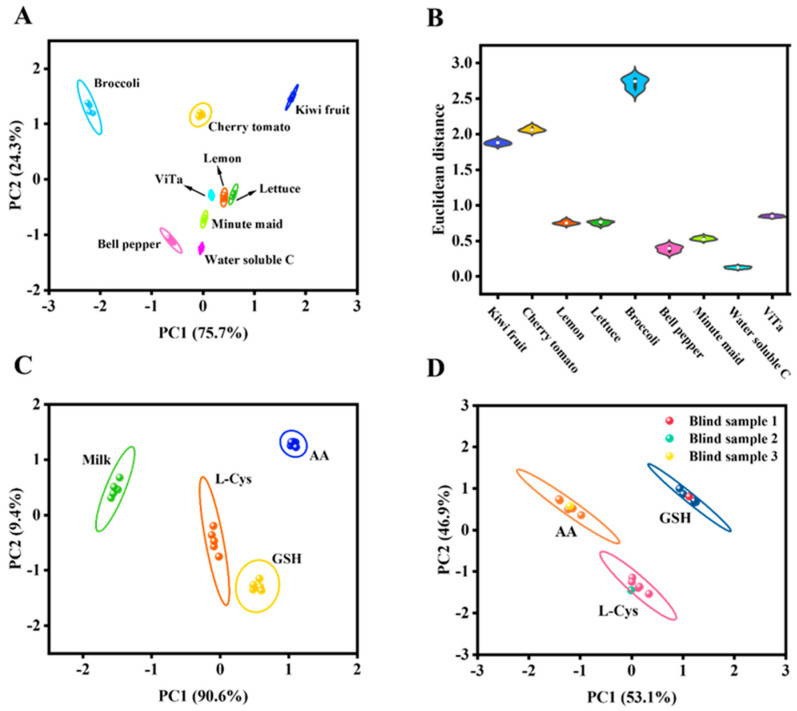
(**A**) PCA plot of the sensor array detecting TAC in fruits, vegetables, and beverages. (**B**) Violin plots for the detection of actual samples. (**C**) Typical score plot for a sensor array used to detect GSH, L-Cys, and AA in milk. (**D**) Plot of PCA score for blind sample determination.

The practical applicability of a sensor array in real-sample detection and analysis is crucial, as it serves as a key criterion for determining its potential utility. In this study, we employed a dual-channel sensor array based on Fe-CDs to evaluate the total antioxidant capacity (expressed in AA equivalents) in various samples. Common fruits (kiwi, cherry tomato, and lemon), vegetables (lettuce, broccoli, and bell pepper), and beverages (Pulpy Orange, Water-Solubilized C, and Vita) were separately analyzed. All prepared sample solutions were diluted 200-fold and filtered through a 0.22 μm filter membrane three times before detection. Additionally, antioxidants (10 μM) were spiked into milk samples to evaluate the feasibility of the sensor array in complex real-world matrices. As shown in Figure 5A, even after a 200-fold dilution, each sample produced a unique response pattern from the sensor array. The PCA plot demonstrated excellent discrimination capability, with all food samples forming distinct clusters showing no overlap. Notably, although lemon and lettuce clusters appeared nearby, they remained completely separated without any overlapping signals. This is because the Fe-CDs dual-channel system captures differential scavenging of •OH/^1^O_2_ by targets, generating distinct UV-vis and FL “fingerprints” that keep clusters separate. The sensor array could accurately detect the intrinsic TAC present in food samples even without antioxidant spiking, as demonstrated by the violin plots (Figure 5B). Figure 5C demonstrated that the milk sample itself generated a weak array signal, while the introduction of three antioxidants resulted in four relatively distinct confidence ellipses. As shown in Figure 5D, the sensor array successfully classified three blind samples into their corresponding regions with high accuracy. These results convincingly demonstrated that the developed sensor array could be directly applied for TAC detection in food samples, showing promising potential for real-time food safety monitoring.

## 4. Conclusions

This study successfully synthesized Fe-CDs with outstanding peroxidase-like activity using a low-cost, economical hot-water method, employing waste coffee grounds as a green precursor. The resulting Fe-CDs exhibit satisfactory fluorescence quantum yields, outperforming most reported biomass-derived carbon dots. Iron doping in Fe^2+^/Fe^3+^ forms within the carbon matrix creates critical Fe–O active sites, endowing Fe-CDs with high substrate affinity. This material catalyzes the conversion of H_2_O_2_ into reactive oxygen species such as •OH and ^1^O_2_. A dual-channel colorimetric/fluorescence sensor array constructed based on this material enables precise identification and detection of AA, GSH, L-Cys, and their mixtures. The sensor demonstrates excellent performance in real samples (vegetables, fruits, beverages). This study not only achieves high-value utilization of waste coffee grounds but also provides an innovative and economical solution for rapid, reliable detection of total antioxidant capacity in food through a single-material, dual-channel sensing strategy.

## Data Availability

The original contributions presented in the study are included in the article/Supplementary Material. Further inquiries can be directed to the corresponding authors.

## Supplementary Materials

The following supporting information can be downloaded at https://www.mdpi.com/article/10.3390/foods14223922/s1. Figure S1. HRTEM images and lattice fringes of Fe-CDs. Figure S2. Correction plots of fluorescence integral area against absorbance for (A) quinine sulfate solution and (B) Fe-CDs solution. Figure S3. (A) FTIR spectra of p-CDs prepared without Fe doping. (B) UV-visible absorption diagram of p-CDs mixed with H_2_O_2_ and TMB. Figure S4. Optimization of reaction conditions from (A) pH, (B) temperature, and (C) time for the determination of the enzymatic kinetic activity of Fe-CDs. Figure S5. (A) The corresponding double reciprocal plots in H_2_O_2_ substrate. (B) Steady-state kinetic assays of Fe-CDs with H_2_O_2_ as substrate. (C) The corresponding double reciprocal plots in the TMB substrate. (D) Steady-state kinetic assays of Fe-CDs with TMB as substrate. Figure S6. Mechanism of fluorescence intensity quenching of Fe-CDs + H_2_O_2_ + TMB by antioxidants (GSH as an example). Figure S7. (A) Fluorescence spectra of p-CDs. (B) Effect of GSH, AA, and L-Cys at the same concentration on the fluorescence intensity of p-CDs. Figure S8. FTIR spectra of p-CDs before reaction (a), and after reaction with three substances, GSH (b), AA (c), and L-Cys (d), respectively. Figure S9. Score plot of the sensor array towards three antioxidants at (A) 1 μM, (B) 10 μM, (C) 25 μM, (D) 50 μM, and (E) 100 μm. Figure S10. Heat map of binary mixtures (A. GSH/L-Cys = 1:1, B. GSH/AA = 1:1, C. AA/L-Cys = 1:1) and concentrations of AA, GSH, and L-Cys at 50 μM. Figure S11. Heat map of binary mixtures (A. GSH/L-Cys/AA = 1:1:2, B. GSH/L-Cys/AA = 1:2:1, C. GSH/L-Cys/AA = 2:1:1) and concentrations of AA, GSH, L-Cys at 50 μM. Table S1. Comparison of QY of coffee ground-derived Fe-CDs with other biowaste-derived carbon dots. Table S2. Comparison of kinetic parameters of Fe-CDs nanozyme with other nanozymes.
