# Supplementary material for: Sustainable Conversion of Coffee Ground Waste into Carbon Dots for Sensing Food Antioxidants
Source: Foods. 2025 Nov 17;14(22):3922. doi: 10.3390/foods14223922 (PMC12651370; doi:10.3390/foods14223922)
Supplement: Supplementary file 1 [file foods-14-03922-s001.zip › foods-3901796-supplementary.pdf]

-Supporting Information-

Low-cost carbon dots sensor array based on waste coffee grounds for accurate identification of total antioxidant capacity in food

Nan Jiang[1], Yuanjing Tao[2,3,*], Ruihong Wang[1], Xiaoran Zhao[1], Jingxuan Ren[1], Chenyang Jiang[4], Zihao Xu[1], Xuming Zhuang[1], Chao Shi[1,*]

[1] School of Chemistry and Chemical Engineering, Yantai University, Yantai 264005, China.

[2] Shandong Dyne Marine Biopharmaceutical Co., Ltd., Weihai 264300, China.

[3] Shandong Dyne Financial Holding Children's Pharmaceutical Co., Ltd., Weihai 264300, China.

[4] Weihai Institute for Food and Drug Control, Weihai 264200, China.

*Corresponding authors: taoyuanjing@dynemed.com; shichao@ytu.edu.cn

Table of Contents

Figure S1. HRTEM images and lattice fringes of Fe-CDs.

Figure S2. Correction plots plots of fluorescence integral area against absorbance for (A) quinine sulphate solution and (B) Fe-CDs solution.

Figure S3. (A) FTIR spectra of p-CDs prepared without Fe doping. (B) UV-visible absorption diagram of p-CDs mixed with H_2O_2 and TMB.

Figure S4. Optimization of reaction conditions from (A) pH, (B) temperature, and (C) time for the determination of the enzymatic kinetic activity of Fe-CDs.

Figure S5. (A) The corresponding double reciprocal plots in H_2O_2 substrate. (B) Steady-state kinetic assays of Fe-CDs with H_2O_2 as substrate. (C) The corresponding double reciprocal plots in TMB substrate. (D) Steady-state kinetic assays of Fe-CDs with TMB as substrate.

Figure S6. Mechanism of fluorescence intensity quenching of Fe-CDs + H_2O_2 + TMB by antioxidants (GSH as an example).

Figure S7. (A) Fluorescence spectra of p-CDs. (B) Effect of GSH, AA, and L-Cys at the same concentration on the fluorescence intensity of p-CDs.

Figure S8. FTIR spectra of p-CDs before reaction (a), and after reaction with three substances, GSH (b), AA (c), and L-Cys (d), respectively.

Figure S9. Score plot of the sensor array towards three antioxidants at (A) 1 μM, (B) 10 μM, (C) 25 μM, (D) 50 μM and (E) 100 μM

Figure S10. Heat map of binary mixtures (A. GSH: L-Cys=1:1, B. GSH: AA=1:1, C. AA: L-Cys=1:1) and concentrations of AA, GSH, L-Cys at 50 μM.

Figure S11. Heat map of binary mixtures (A. GSH: L-Cys: AA=1:1:2, B.GSH: L-Cys: AA=1:2:1, C. GSH: L-Cys: AA=2:1:1) and concentrations of AA, GSH, L-Cys at 50 μM.

Table S1. Comparison of QY of coffee ground-derived Fe-CDs with other biowaste-derived carbon dots.

Table S2. Comparison of kinetic parameters of Fe-CDs nanozyme with other nanozymes.

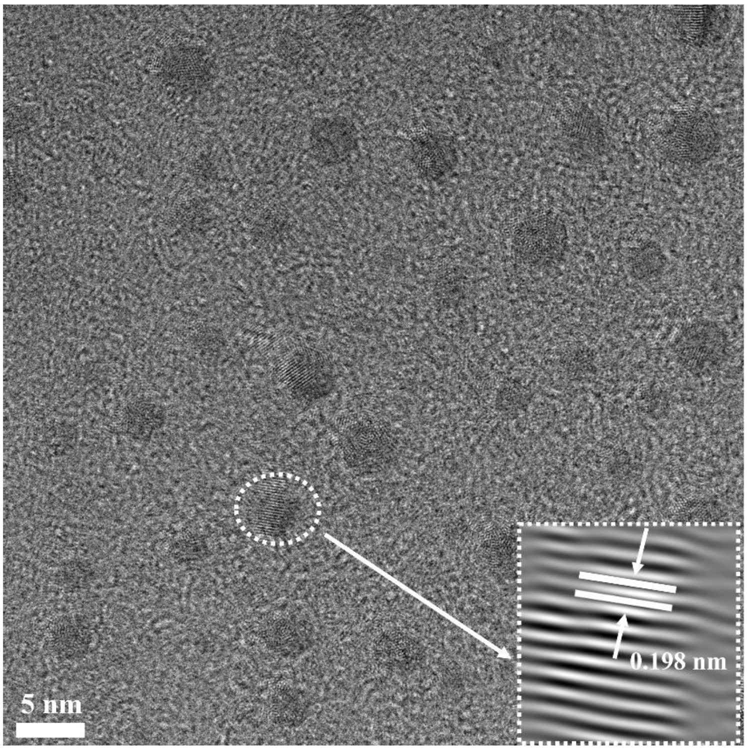

Figure S1. HRTEM images and lattice fringes of Fe-CDs.

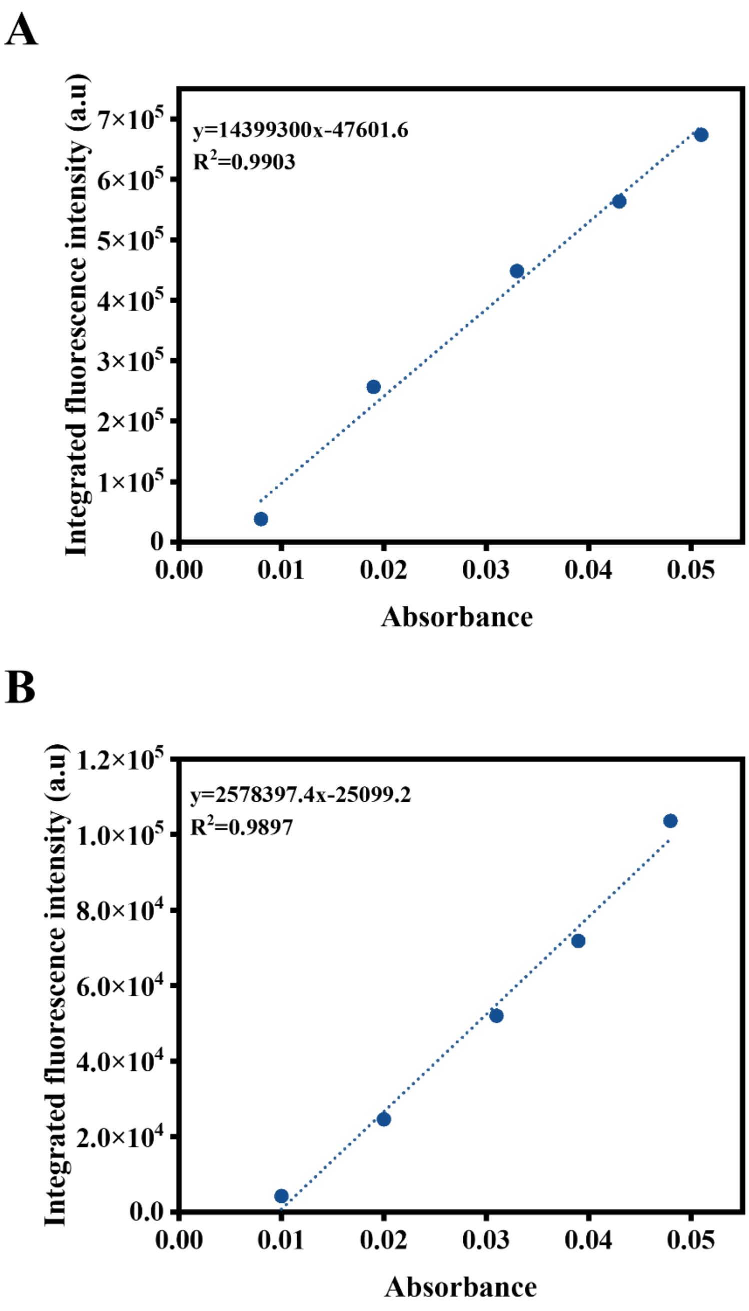

Figure S2. Correction plots of fluorescence integral area against absorbance for (A) quinine sulphate solution and (B) Fe-CDs solution.

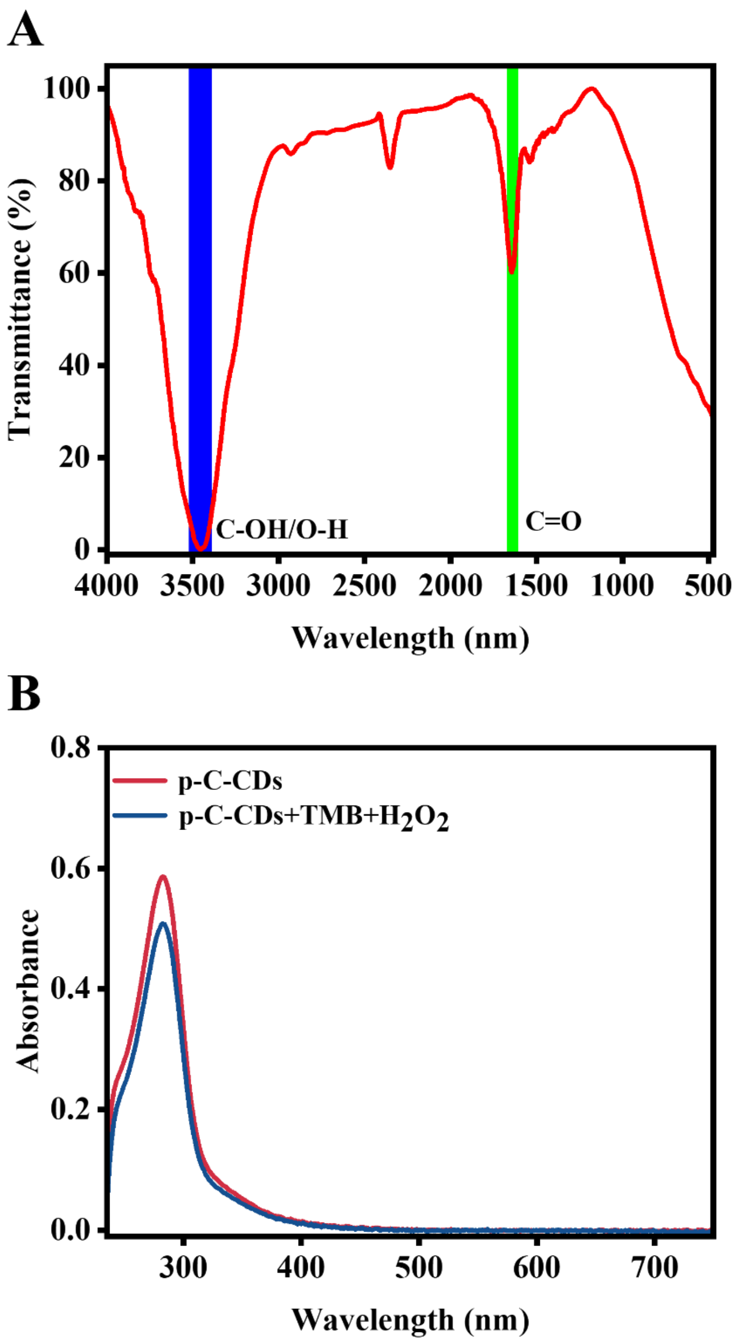

Figure S3. (A) FTIR spectra of p-CDs prepared without Fe doping. (B) UV-visible absorption diagram of p-CDs mixed with H_2O_2 and TMB.

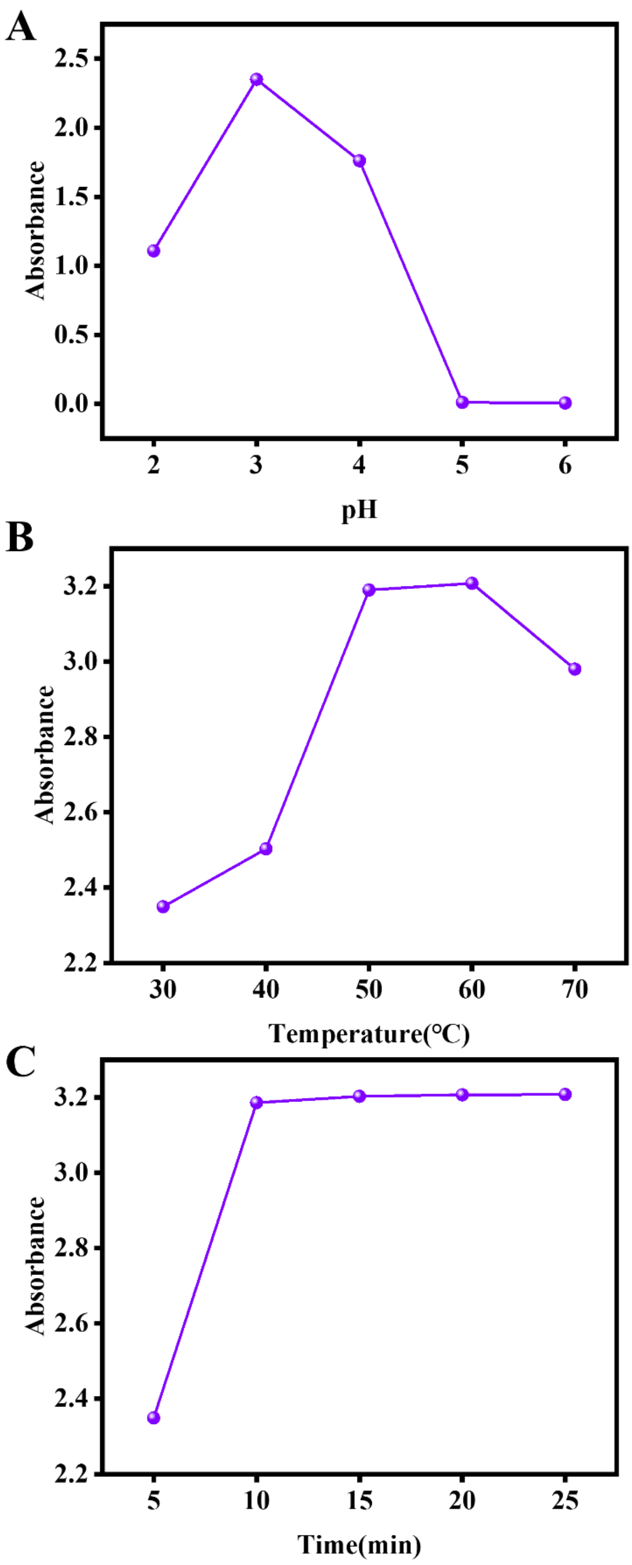

Figure S4. Optimization of reaction conditions from (A) pH, (B) temperature, and (C) time for the determination of the enzymatic kinetic activity of Fe-CDs.

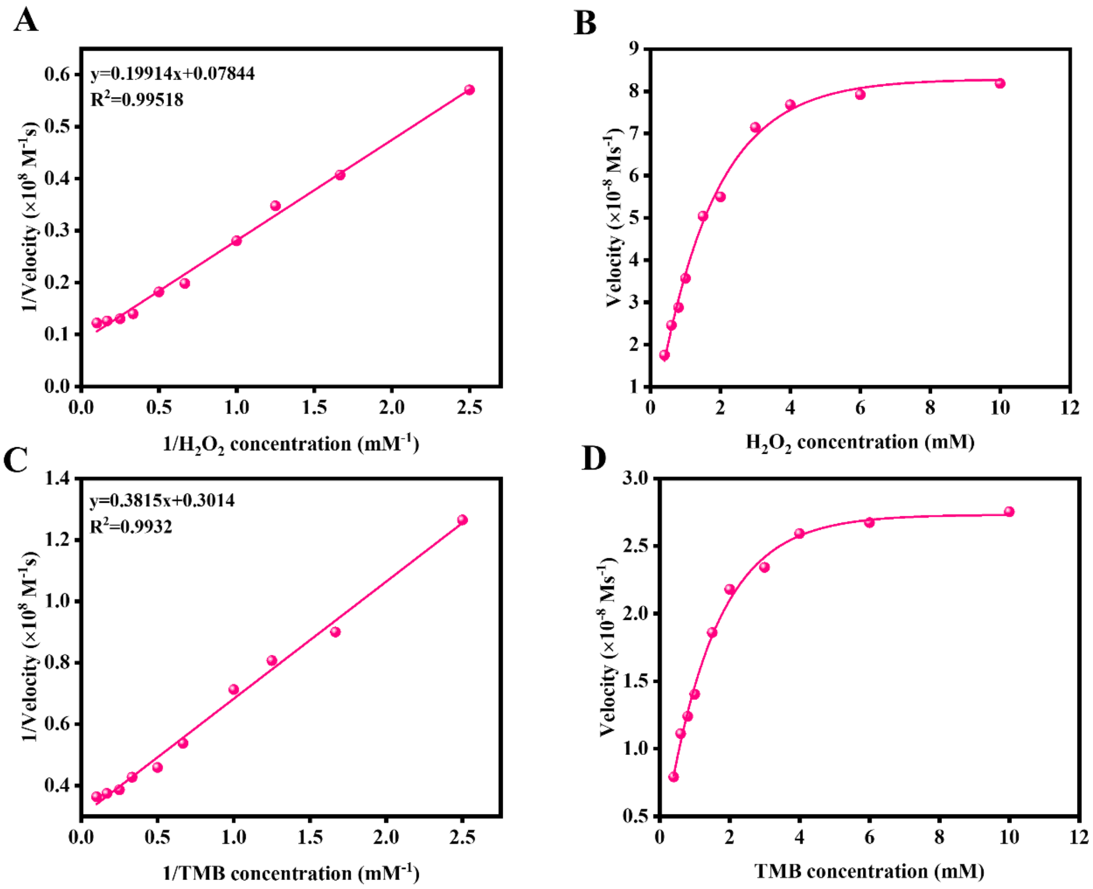

Figure S5. (A) The corresponding double reciprocal plots in H_2O_2 substrate. (B) Steady-state kinetic assays of Fe-CDs with H_2O_2 as substrate. (C) The corresponding double reciprocal plots in TMB substrate. (D) Steady-state kinetic assays of Fe-CDs with TMB as substrate.

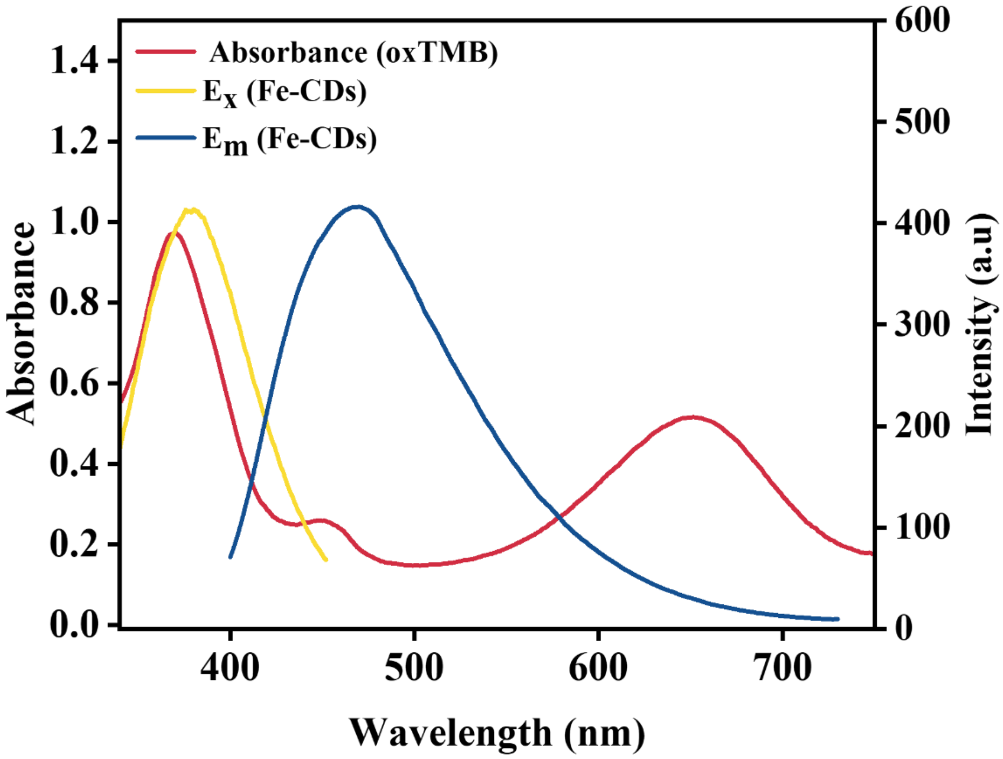

Figure S6. Mechanism of fluorescence intensity quenching of Fe-CDs + H_2O_2 + TMB by antioxidants (GSH as an example).

A

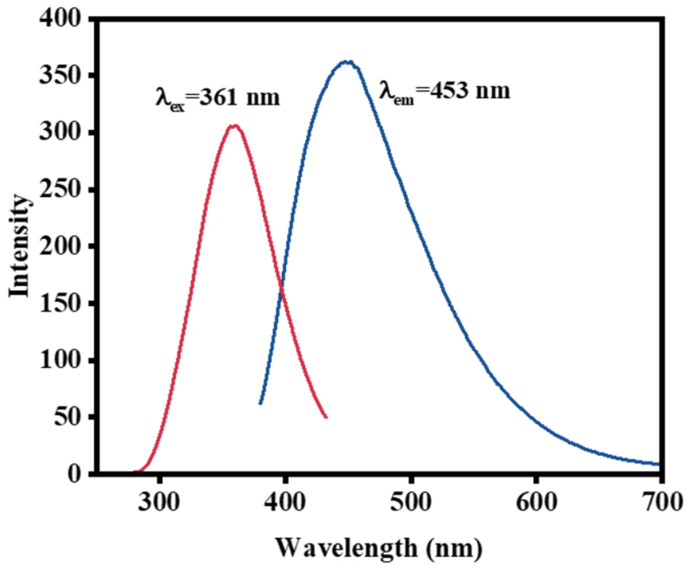

B

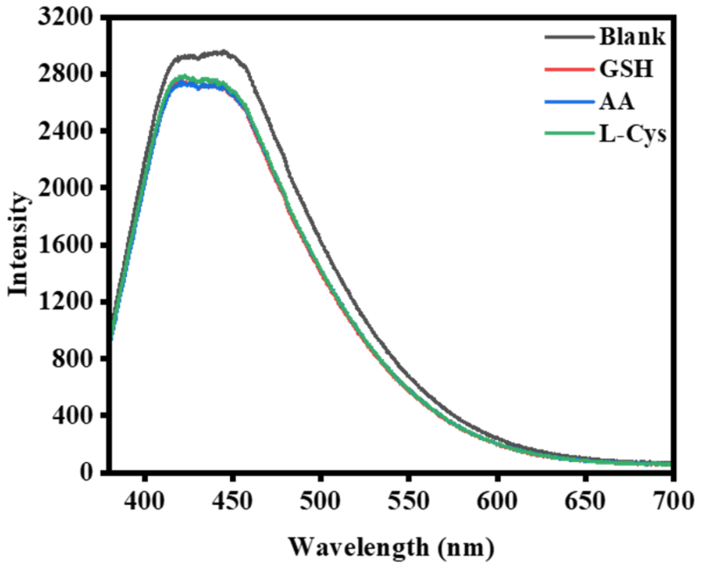

Figure S7. (A) Fluorescence spectra of p-CDs. (B) Effect of GSH, AA, and L-Cys at the same concentration on the fluorescence intensity of p-CDs.

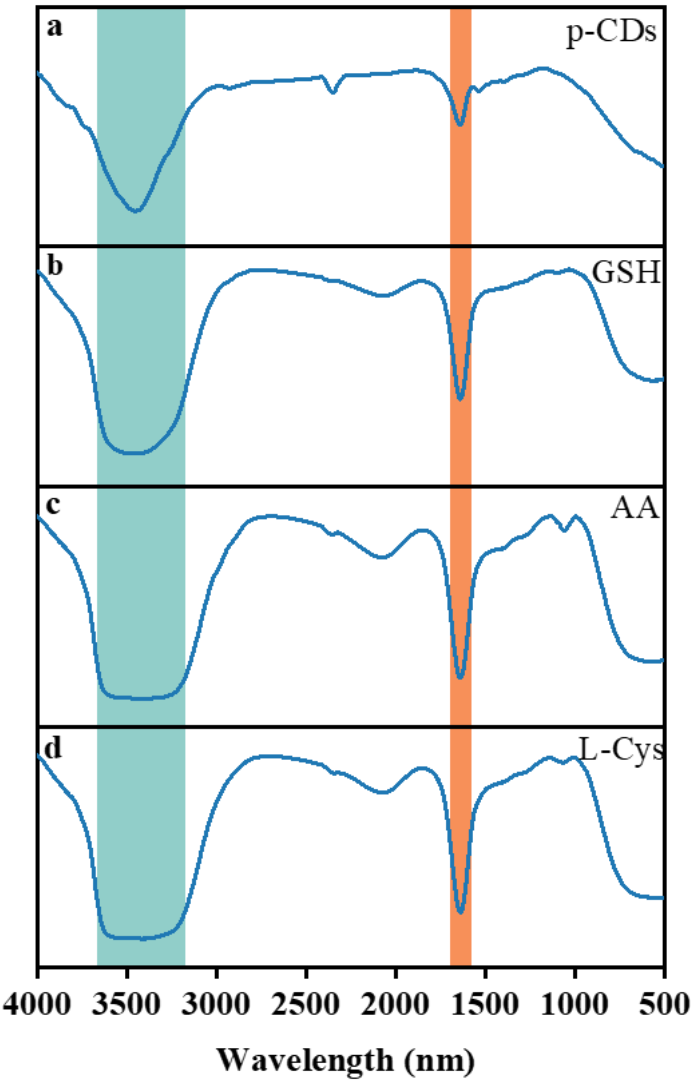

Figure S8. FTIR spectra of p-CDs before reaction (a), and after reaction with three substances, GSH (b), AA (c), and L-Cys (d), respectively.

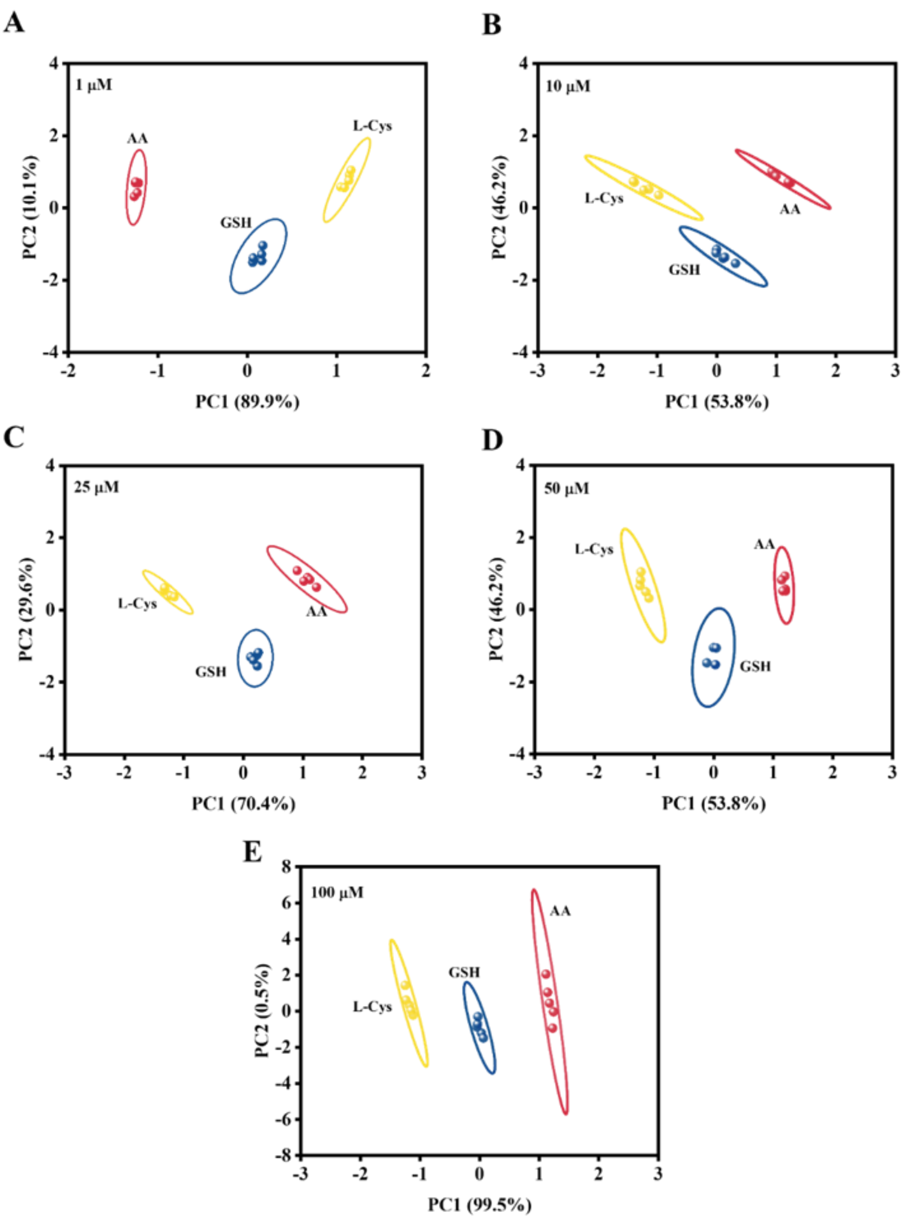

Figure S9. Score plot of the sensor array towards three antioxidants at (A) 1 μM, (B) 10 μM, (C) 25 μM, (D) 50 μM and (E) 100 μM.

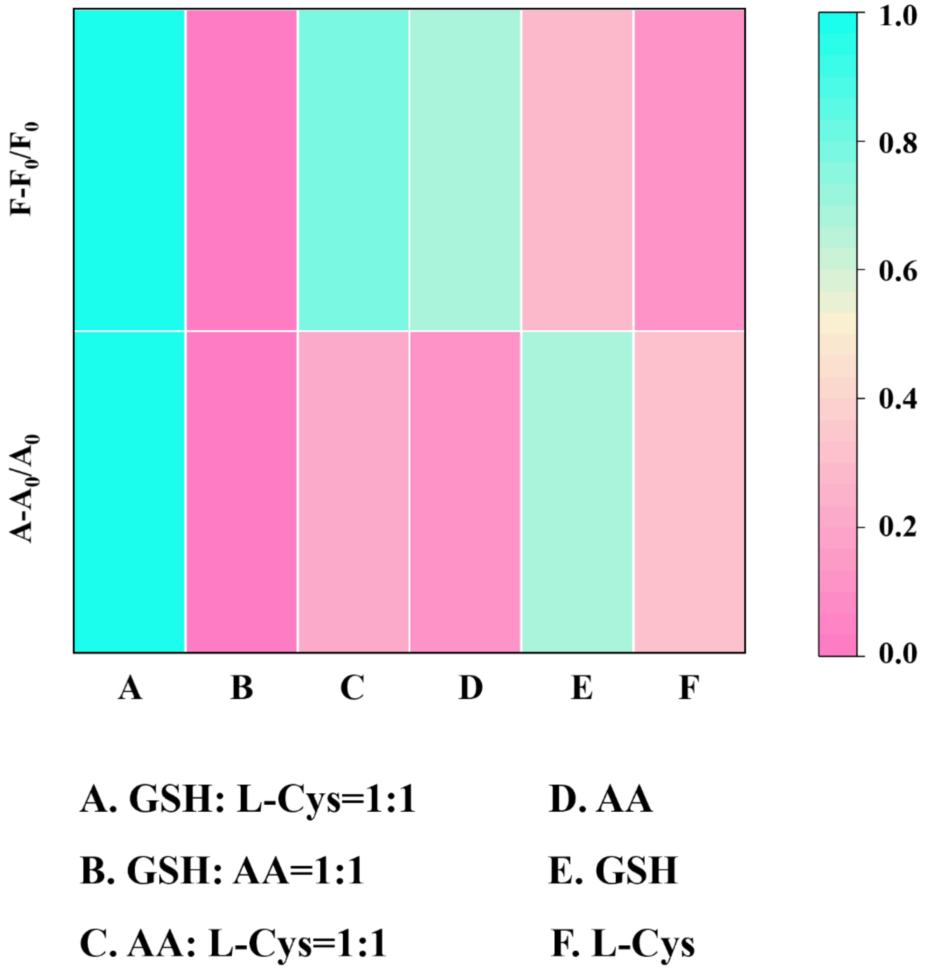

Figure S10. Heat map of binary mixtures (A. GSH:L-Cys=1:1, B. GSH: AA=1:1, C. AA: L-Cys=1:1) and concentrations of AA, GSH, L-Cys at 50 μM.

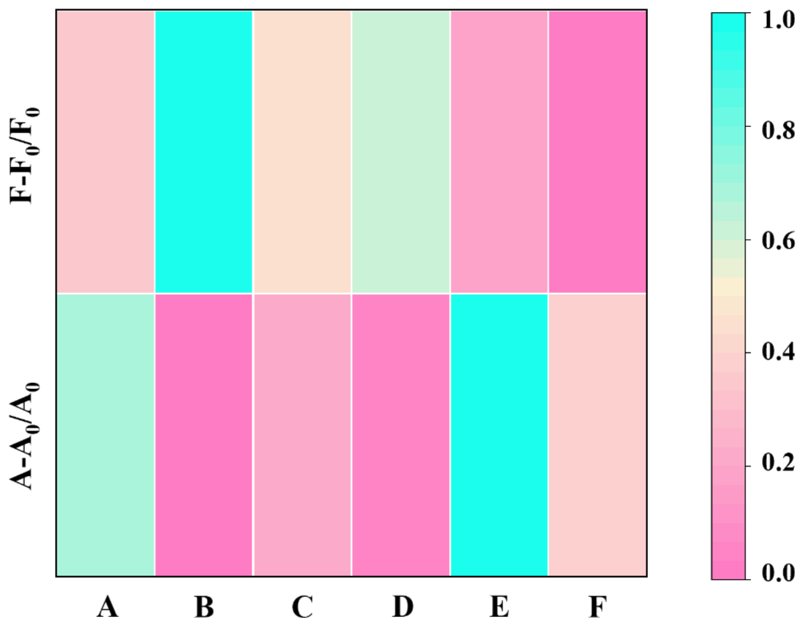

A. GSH: L-Cys: AA=1:1:2 **D. AA**

B. GSH: L-Cys: AA =1:2:1 **E. GSH**

C. GSH: L-Cys: AA =2:1:1 **F. L-Cys**

Figure S11. Heat map of binary mixtures (A. GSH: L-Cys: AA=1:1:2, B.GSH: L-Cys: AA=1:2:1, C. GSH: L-Cys: AA=2:1:1) and concentrations of AA, GSH, L-Cys at 50 μM.

Table S1. Comparison of QY of coffee ground-derived Fe-CDs with other biowaste-derived carbon dots.

Carbon dots	Waste biomass	QY (%)	Ref.
EBCDs	Bee pollen	4.8	Chin. Chem. Lett. 2022, 33, 2942
CA-CFCDs	Coffee grounds	3.7	J. Clean. Prod. 2023, 392136250
CQDs	Cassava stem	1.66	Cellulose 2021, 29, 367
LLCDs	Lemon leaves	7.2	Spectrochim. Acta A 2023, 286, 122024
MF-CQDs	Magnolia flower	8.13	Mater. Res. Bull. 2020, 124, 110730
CDs	Camellia oleifera	2.98	Chem. Pap. 2023, 78, 1145
Fe-CDs	Coffee grounds	4.18	Food Chem. 2023, 429, 136957
Fe-CDs	Coffee grounds	9.18	This work

The abbreviations and full names of each material in Table S1 are as follows:

EBCDs (carbon dots prepared from bee pollen extracted with ethanol),

CA-CFCDs (carbon dots prepared from spent coffee grounds and functionalised with carboxylic acids),

CQDs (green quantum dots from cassava stem),

LLCDs (carbon dots from wild lemon leaves),

MF-CQDs (carbon quantum dots made from magnolia flowers),

CDs (Camellia oleifera shell-derived biomass carbon dots),

Fe-CDs (carbon dots prepared from waste coffee grounds using ferric chloride as a dopant).

Table S2. Comparison of kinetic parameters of Fe-CDs nanozyme with other nanozymes.

Materials	Substrate	K_m (mM)	V_{max} (10^{-8} M•s^{-1})	Ref.
Ag-CQD	TMB	0.0997	0.578	Food Chem. 2024,
	H_2O_2	1.883	1.375	447, 139020
Fe-CDs	TMB	0.028	10.65	Microchem. J. 2024,
	H_2O_2	18.6	12.34	197, 109720
Cu-hemin	TMB	0.56	11.11	Microchim. Acta
	H_2O_2	14.615	6.25	2024, 191, 575
S-CDs/AuNPs	TMB	0.7	0.23	Food Chem. 2024,
	H_2O_2	1.2	1.04	434, 137440
CDs	TMB	2.51	16.7	J. Colloid Interf. Sci.
	H_2O_2	65.98	4.5	2022, 611, 545-553
Fe-CDs	TMB	1.27	3.32	This work
	H_2O_2	2.54	12.76	

The abbreviations and full names of each material in Table S2 are as follows:

Ag-CQD (ultra-trace Ag doped carbon quantum dots),

Fe-CDs (functional carbon dots nanozymes doped Fe),

Cu-hemin (three-dimensional porous bimetallic framework nanozyme constructed from Cu^{2+} and Hemin),

S-CDs/AuNPs (S doped carbon dots modified gold nanoparticles),

CDs (metal free carbon dots),

Fe-CDs (carbon dots prepared from waste coffee grounds using ferric chloride as a dopant).
